# Exploring the therapeutic potential of *Quercus ilex* acorn extract in papillomavirus-induced lesions

**DOI:** 10.14202/vetworld.2024.2644-2658

**Published:** 2024-11-28

**Authors:** Beatriz Medeiros-Fonseca, Ana I. Faustino-Rocha, Maria João Pires, Maria João Neuparth, Helena Vala, Cármen Vasconcelos-Nóbrega, Irene Gouvinhas, Ana Novo Barros, Maria Inês Dias, Lillian Barros, Margarida M. S. M. Bastos, Lio Gonçalves, Luís Félix, Carlos Venâncio, Rui Medeiros, Rui Miguel Gil da Costa, Paula A. Oliveira

**Affiliations:** 1 Center for the Research and Technology of Agro-Environmental and Biological Sciences (CITAB), University of Trás-os-Montes e Alto Douro, 5000-801 Vila Real, Portugal; 2 Institute for Innovation, Capacity Building and Sustainability of Agri-food Production (Inov4Agro), University of Trás-os-Montes e Alto Douro, 5000-801 Vila Real, Portugal; 3 Molecular Oncology and Viral Pathology Group, Research Center of IPO Porto (CI-IPOP)/RISE@CI-IPOP (Health Research Network), Portuguese Oncology Institute of Porto (IPO Porto), Porto Comprehensive Cancer Center (Porto. CCC), 4200-072 Porto, Portugal; 4 Department of Zootechnics, School of Sciences and Technology, University of Évora, 7000-812 Évora, Portugal; 5 Comprehensive Health Research Center, University of Évora, 7000-812 Évora, Portugal; 6 Department of Veterinary Sciences, University of Trás-os-Montes e Alto Douro, 5000-801 Vila Real, Portugal; 7 Laboratory for Integrative and Translational Research in Population Health (ITR), Research Center in Physical Activity, Health and Leisure (CIAFEL), Faculty of Sports, University of Porto, 4200-450 Porto, Portugal; 8 UCIBIO-Applied Molecular Biosciences Unit, Translational Toxicology Research Laboratory, University Institute of Health Sciences (1H-TOXRUN, IUCS-CESPU), 4585-116 Gandra, Portugal; 9 CERNAS-IPV Research Center, Polytechnique Institute of Viseu, 3504-510 Viseu, Portugal; 10 Polytechnique Institute of Viseu, Agrarian School of Viseu, Campus Politécnico 3504-510 Viseu, Portugal; 11 The Mountain Research Center of the Polytechnic Institute of Bragança (CIMO), Associate Laboratory for Sustainability and Technology in Mountain Regions (LA SusTEC), Instituto Politécnico de Bragança, Santa Apolónia Campus, 5300- 253 Bragança, Portugal; 12 Laboratory for Process Engineering, Environment, Biotechnology and Energy (LEPABE), Faculty of Engineering of the University of Porto (FEUP), 4200-465 Porto, Portugal; 13 Associate Laboratory in Chemical Engineering (ALiCE), Faculty of Engineering of the University of Porto (FEUP), 4200-465 Porto, Portugal; 14 Department of Engineering, University of Trás-os-Montes e Alto Douro, 5000-801 Vila Real, Portugal; 15 Institute for Systems and Computer Engineering, Technology and Science (INESC-TEC), 4200-465 Porto, Portugal; 16 Animal and Veterinary Research Center (CECAV), University of Trás-os-Montes and Alto Douro, 5000-801 Vila Real, Portugal; 17 Department of Animal Science, School of Agrarian and Veterinary Sciences (ECAV), University of Trás-os-Montes e Alto Douro, Vila Real, Portugal; 18 Department of Research, Portuguese League against Cancer, Regional Nucleus of the North (LPCC-NRN), 4200-177 Porto, Portugal; 19 Department of Biomedicine, Faculty of Medicine, University of Porto, 4200-319 Porto, Portugal; 20 Virology Service, Portuguese Institute of Oncology (IPO), 4200-072 Porto, Portugal; 21 Biomedical Research Center (CEBIMED), Faculty of Health Sciences of Fernando Pessoa University (UFP), 4249-004 Porto, Portugal; 22 Postgraduate Program in Adult Health (PPGSAD), Department of Morphology, Federal University of Maranhão (UFMA), São Luís 65020-070, Brazil

**Keywords:** acorn, antioxidant capacity, cancer, mouse model, polyphenols, *Quercus* spp

## Abstract

**Background and Aim:**

Papillomaviruses (PVs) infections have been documented in numerous animal species across different regions worldwide. They often exert significant impacts on animal health and livestock production. Scientists have studied natural products for over half a century due to their diverse chemical composition, acknowledging their value in fighting cancer. Acorns (*Quercus ilex*) are believed to have several unexplored pharmacological properties. This study aimed to evaluate the *in vivo* safety and cancer chemopreventive activity of an infusion extract of *Q. ilex* in a transgenic mouse model of human PV (HPV)-16, which developed squamous cell carcinomas through a multistep process driven by HPV16 oncogenes.

**Materials and Methods:**

*Q. ilex* extract was prepared by heating in water at 90°C and then characterized by mass spectrometry. Phenolic compounds from this extract were administered in drinking water to female mice in three different concentrations (0.03, 0.06, and 0.09 g/mL) over a period of 28 consecutive days. Six groups (n = 6) were formed for this study: group 1 (G1, wildtype [WT], water), group 2 (G2, HPV, water), group 3 (G3, WT, 0.09 g/mL), group 4 (G4, HPV, 0.03 g/mL), group 5 (G5, HPV, 0.06 g/mL), and group 6 (G6, HPV, 0.09 g/mL). Throughout the experiment, humane endpoints, body weight, food intake, and water consumption were recorded weekly. Following the experimental period, all mice were sacrificed, and blood, internal organs, and skin samples were collected. Blood was used to measure glucose and microhematocrit and later biochemical parameters, such as creatinine, urea, albumin, alanine aminotransferase, and total proteins. Histological analysis was performed on skin and organ samples.

**Results:**

The administration of *Q. ilex* extract resulted in a statistically significant increase in relative organ weight among HPV transgenic animals, indicating adaptive biological response to the tested concentrations. Moreover, a reduction in characteristic skin lesions was observed in animals treated with the 0.06 and 0.09 g/mL extract.

**Conclusion:**

These results provide a favorable chemopreventive profile for *Q. ilex* extract at concentrations of 0.06 and 0.09 g/mL. This study highlights the potential of *Q. ilex* extract as a safe and effective therapeutic strategy against HPV16-associated lesions in transgenic mouse models. The limitation of our study was the durability of transgenic animals. As a more sensitive species, we must always be careful with the durability of the test. We intend to study concentrations of 0.06 and 0.09 g/mL for longer to further investigate their possible effects.

## Introduction

Papillomaviruses (PVs) are related to epithelial malignancies in animals, including cancer in humans [1, 2]. PVs are characterized as small, non-enveloped deoxyribonucleic acid (DNA) viruses known to induce lesions on the skin (warts) and mucous membranes (condylomas) across various species. PVs exhibit species specificity, with infections reported in mammals, birds, fish, and reptiles. They demonstrate either tropism for the skin or keratinized mucosae or dual tropism for epithelial tissues [3]. Canine PVs (CPVs) are associated with exophytic papillomas, endophytic papillomas, and squamous cell carcinomas [4]. These lesions are often transient, whereas others persist and potentially advance. Many documented cases are characterized as CPV-1 [5]. Bovine PVs (BPVs) are prevalent worldwide in bovine populations [3] and have provided important lessons for virology, as previously reported by Gil da Costa and Medeiros [6]. BPV-1 and BPV-2 are the most commonly encountered types of BPVs, and they are associated with both papillomas and fibropapillomas, often involving the dermis [7]. BPVs can cross interspecies boundaries, warranting significant attention [8]. Viral DNA is detected in healthy animals in a latent form, whereas in sick animals, it is found in lymph nodes, blood, sperm, saliva, urine, placenta, fetuses, and milk after post-pasteurization [9–14]. Understanding the potential for transmission to humans is crucial to safeguard public and animal health [7]. In humans, human PV (HPV)-16 and HPV-18, the two most prevalent high-risk strains in humans, account for approximately 70% of all HPV-related cervical and head and neck cancers [15, 16]. PV infections have been documented in numerous animal species across different regions worldwide [17]. They often significantly impact animal health and livestock production, directly influencing local economies and potentially affecting human health indirectly [18]. Therefore, it is crucial to explore sustainable preventive measures that leverage the potential offered by natural resources [19]. Thus, for more than half a century, scientists have investigated natural products due to the extraordinary chemical diversity found in nature [20]. Natural products are widely regarded as a valuable reservoir of bioactive compounds with significant therapeutic potential for fighting cancer [19–21].

Acorns, the fruit of oak trees (*Quercus* spp.), belonging to the *Fagaceae* family, have long been recognized for their high nutritional value and versatile uses in various culinary applications, including the production of flour, bread, and jam [22–26]. Numerous research studies have demonstrated that leaves and seed of *Quercus ilex* have medicinal properties such as antioxidant [23], antibacterial [24], antitumor [25], and antidiabetic [27]. Alkaloids, glycosides, tannins, and phenolic compounds, such as flavonoids, resins, saponins, terpenes, and steroids, are the main bioactive components of *Quercus* spp. acorns associated with their pharmacological properties [28, 29]. Acorns are rich in essential amino acids, fatty acids, vitamins, polyphenols, and minerals, contributing to their well-established nutritional, functional, and health benefits. In addition, it is a gluten-free source that can be efficiently used to develop gluten-free products [27, 30]. *In vitro* studies utilizing *Quercus* spp. extracts showed notable antiproliferative activity, triggering early apoptotic cell death in cancer cell lines such as HeLa (Henrietta Lacks) (HPV18-positive cervical carcinoma) and AGS (human gastric carcinoma) [28]. Considering these promising *in vitro* findings, conducting *in vivo* studies is imperative to comprehensively understand the biological effects of acorn extracts and assess their potential as anticancer agents. Thus, due to the similarities between PVs, such as their structure and behavior, the specific affinity for epithelial tissues that tend to cause damage to the skin and mucous membranes of their hosts [31], and the availability of this strain in our laboratory, we decided to use mice genetically modified for K14-HPV16 [32].

This study aimed to examine the *in vivo* effects of *Q. ilex* extract in a transgenic mouse model of high-risk HPV type 16 (HPV16). This mouse model carried the complete HPV16 early region and developed pre-malignant and malignant lesions at multiple sites [32]. Moreover, the HPV16 transgenic animals were predisposed to develop liver and spleen inflammation [33]. This model was previously employed to evaluate the anticancer or pro-cancer effects of natural products, including ptaquiloside from *Pteridium* spp. (bracken fern) [34], dietary polyphenols such as curcumin and rutin [33], and extracts from *Laurus nobilis* (laurel) [35]. Therefore, this model serves as a valuable tool for assessing the potential of natural products to intervene in carcinogenesis and mitigate or intensify these inflammatory phenomena, providing essential toxicological safety data. In this study, an aqueous acorn extract was administered to both HPV16 transgenic and matched wild-type (WT) mice to assess its potential for cancer chemoprevention and its toxicological profile.

## Materials and Methods

### Ethical approval

The study was approved by the Authorities Responsible for Animal Welfare of the University of Trás-os-Montes and Alto Douro (UTAD) (approval no.852-e-CITAB-2020_A_1-e-CITAB-2021) and by the Portuguese Veterinary Directorate (approval no.014139) and was carried out at the UTAD animal facility. Our study followed the ARRIVE guidelines. According to Medeiros-Fonseca *et al*. [35], this study used the minimum number of animals necessary to ensure robust results. All animals were housed under species-appropriate conditions and handled by experienced personnel. The control and K14HPV16 animals were randomly assigned to experimental groups to minimize bias.

### Study period and location

The study was conducted during November and December 2021 at the animal facilities of the UTAD, located in Vila Real, Portugal.

### Sample preparation

The samples of *Q. ilex* were obtained from Bolota Viva (E210409), which were harvested between November and December 2020 and characterized by Bolota Viva as *Q. ilex*. Bolota Viva exclusively practices organic production in the Montado Alentejano region in Portugal.

*Q. ilex* extract was prepared through infusion extraction. Three concentrations (0.03, 0.06, and 0.09 g/mL) of *Q. ilex* extract were prepared in tap water (heated to 90°C for 5 min) in a French press, then filtered, and allowed to cool at room temperature (20–25°C) for 20 min. We used tap water to replicate the conditions under which tea is prepared in real life. Tap water is more accessible, making it easier for anyone to replicate the treatment and apply it to their animals.

### Phenolic compound profile and antioxidant capacity

The total phenolic, ortho-diphenol, and flavonoid contents of the *Q. ilex* extract were determined by spectrophotometric methods according to the description by Gouvinhas *et al*. [36]. In addition, the aqueous extract was prepared at 10 mg/mL and analyzed using liquid chromatography coupled to diode array detection and electrospray ionization mass spectrometry system to determine the chemical profile [37]. The antioxidant capacity of the *Q. ilex* extract was determined by ferric reducing antioxidant power spectrophotometric method, as previously reported by Mena *et al*. [38].

### Animals

Thirty-six female *Mus musculus* of the FVB/n strain were used: 24 transgenic (HPV+) and 12 (HPV–, WT) mice aged 20–23 weeks. This is the age at which cutaneous lesions in this mouse strain begin to progress from the hyperplastic to dysplastic stage, affording an opportunity to experimentally prevent this progression [32]. Female mice were chosen to reduce the aggressive behavior of the animals, which is common in males [39]. The mouse strain was donated by Drs. Jeffrey Arbeit and Douglas Hanahan (University of California) through the National Cancer Institute Mouse Repository [40]. The animals were genotyped using a polymerase chain reaction, as previously described by Paiva *et al*. [41].

### Experimental design

Mice were acclimatized for a week and housed under controlled experimental conditions, including temperature (23°C ± 2°C), light-dark cycle (light between 8:00 and 20:00 h), and relative humidity (50% ± 10%). The mice had *ad libitum* access to standard chow (4RF21 GLP, Mucedola, Italy) and tap water throughout the experiment. A power analysis [35], ethical considerations, and specific requirements were considered to determine the appropriate number of animals for the trial. Mice were randomly divided into six groups: group 1 (G1, WT, water, n = 6), group 2 (G2, HPV, water, n = 6), group 3 (G3, WT, 0.09 g/mL, n = 6), group 4 (G4, HPV, 0.03 g/mL n = 6), group 5 (G5, HPV, 0.06 g/mL, n = 6), and group 6 (G6, HPV, 0.09 g/mL, n = 6). The animals’ weights, food, and drinks were recorded every 8 days. Humane endpoints were evaluated on a weekly basis, assessing parameters such as body mass, fur condition, eye and ear health, whisker appearance, mental alertness, and presence of papilloma, as outlined by Silva-Reis *et al*. [42]. Therefore, if any animal reaches a score of four, it is euthanized before the end of the experiment [43]. *Q. ilex* extract was administered in drinking water for 28 days, with the water being renewed every two consecutive days. No significant solubility or precipitation problems were observed throughout the study. Controls were implemented to monitor any changes in the quality of the solution and ensure the compound’s effectiveness. At the end of the study, all animals were euthanized through intraperitoneal injection using a 10:1 ratio of ketamine (100 mg/kg) to xylazine (10 mg/kg), followed by exsanguination through cardiac puncture, in agreement with the guidelines established by the Federation of European Laboratory Animal Science Associations (FELASA) [44]. Blood and organ samples were subsequently collected for analysis.

### Microhematocrit and biochemical parameters

Blood samples were centrifuged using a Pro-Vet centrifuge (Centurion Scientific Limited, West Sussex, UK) at 13,500× *g* for 5 min, and plasma was collected for analysis of serum biochemistry. The microhematocrit value was measured using a microhematocrit reader (PrO-Vet HE Haematocrit, United Kingdom). Serum concentrations of albumin, total protein, creatinine, urea, and alanine aminotransferase (ALT) were determined using an autoanalyzer (Prestige 24i, PZ Cormay S.A., Łomianki, Poland). To determine serum glucose levels, the animals were fasted for 12 h. After this period, blood glucose levels were measured in each animal. Glucose levels were measured using a GlucoMen Areo 2 K glucometer with *GlucoMen Areo Sensor* brand strips (A. Menarini Diagnostics, Florence, Italy).

### Histological analysis

Tissue samples were fixed in 10% neutral buffered formalin, transferred to histological cassettes, dehydrated in a graded series of ethanol and xylene, and embedded in paraffin. The samples were then serially sectioned at 4 μm using a microtome, collected on glass slides, and deparaffinized by placing the slides in xylene twice for 5 min. Finally, the samples were hydrated using a graded ethanol series and stained with hematoxylin and eosin using an automated staining apparatus for routine histopathological diagnosis. All slides were examined by light microscopy using a Zeiss microscope. Axioplan 2 and image processing were performed using the LAS Advanced Analysis Software Bundle (No: 12730448, Leica Microsystems, Wetzlar, Germany). In general, ear pavilion and chest skin lesions were classified as dysplastic, papilloma, hyperplastic, and carcinoma, as previously described by Gil da Costa *et al*. [33]. Lung lesions were classified as mononuclear inflammation, atelectasis, emphysema, bleeding, airway desquamation, congestion, hyalinization, and hemorrhage. The liver and spleen were selected for histological observation because these organs were previously described as suffering from severe inflammation in this animal model [35]. Liver lesions were classified as vacuolar, inflammation, and congestion, and spleen lesions were classified as hyperplasia and congestion [33, 35]. Heart lesions were classified as myofiber hyalinization, congestion, hemorrhage, and vascular ectasia, whereas kidney lesions were classified as inflammatory infiltrate [33], hydronephrosis [45], congestion [46], hyperemia [47], and vascular ectasia [48].

### Liver and kidney oxidative stress

Liver and kidney samples were collected and stored at −80°C until they were processed for oxidative stress analysis. To determine oxidative stress parameters, samples were homogenized with cold buffer (0.32 mM of sucrose, 20 mM of HEPES, 1 mM of MgCl_2_, and 0.5 mM of phenylmethyl sulfonylfluoride, pH 7.4) using TissueLyser II (Qiagen, Hilden, Germany) [49]. The homogenate was centrifuged at 15,000× *g* for 20 min at 4°C (PrismR, Labnet International, USA), and the supernatants were collected for analysis. Superoxide dismutase (Cu/Zn-SOD) activity was determined by the nitroblue tetrazolium reduction generated by the xanthine/xanthine oxidase system at 560 nm [50]. SODs from bovine erythrocytes were used to construct a standard curve (0–600 U/mL). Catalase (CAT) activity was determined at 240 nm as described by Aebi [51] and was calculated using bovine catalase as a standard (0–696 U/mL). Glutathione peroxidase (GPx) activity was determined at 340 nm [52], using an extinction coefficient of 6.22 mM^-1^/cm. Glutathione reductase (GR) activity was determined by measuring the increase in nicotinamide adenine dinucleotide phosphate hydrogen (NADPH) absorbance at 340 nm [53] using a molar extinction coefficient of 6.22 mM^-1^/cm. Glutathione S-Transferase (GST) activity was determined by the increase in absorbance at 340 nm due to the conjugation of the thiol group of glutathione (GSH) to the 1-chloro-2,4-dinitrobenzene substrate [54], using a molar extinction coefficient of 9.60 mM^-1^/cm. GSH levels were determined by measuring both the reduced and oxidized glutathione disulfide (GSSG) states using the fluorochrome ortho-phthalaldehyde at 320 and 420 nm for excitation and emission wavelengths, respectively [55]. Concentrations were estimated using GSH and GSSG standard curves (0–1 mM). The ratio of GSH to GSSG was calculated as the oxidative stress index. Malondialdehyde (MDA) content, an indicator of lipid peroxidation (LPO), was measured at wavelengths of 535 nm (MDA-thiobarbituric acid [TBA] adducts) and 600 nm (nonspecific adducts) using the TBA-based method described elsewhere [56]. MDA was estimated from a standard curve (0–1000 μM) of malonaldehyde bis (dimethyl acetal). Carbonyls were estimated using the method described by Mesquita *et al*. [57] and were determined at 450 nm.

### Statistical analysis

Statistical analysis was conducted using IBM SPSS Statistics for Windows, version 26 (IBM Corp., Armonk, N.Y., USA), and GraphPad Prism version 9 (Boston, Massachusetts, USA). Normal distribution was assessed using the Shapiro–Wilk test, and homogeneity of variance was evaluated using the *Levene* test. For data with a normal distribution, analysis of variance (ANOVA) followed by the *Bonferroni* test was applied. Non-parametric tests, such as the Kruskal–Wallis test, were used for data that did not conform to a normal distribution. After the Kruskal–Wallis test, we performed pairwise comparisons that included Dunn’s *post hoc* test with Bonferroni correction to identify which groups were significantly different from each other. Statistical significance was defined as p < 0.05. Parameters subjected to this criterion included body weight, food and drink consumption, organ mass, humane endpoints, and hematological and oxidative stress parameters. The association between histological lesions and groups was examined using the Chi-square test.

- Humane endpoints = (sum score *per* animal)/(number of animals *per* cage).
- Weight gain = (final weight–initial weight)/(initial weight) × 100 (%).
- Average daily consumption (drink/food) *per* animal *per* day = (initial weight) – (final weight)/(number of days between weighings) × (number of animals *per* cage) (g). We did not use metabolic cages; the animals in each group were kept in the same cage. Thus, because the animals were in the same cage, for animal welfare reasons, we calculated the average food and water consumption per animal.
- Relative weight of organs (%) = (organ weight, g)/(body weight, g) × 100 (%).

## Results

### Profile of phenolic compounds and antioxidant capacity of aqueous extracts

The chemical contents of the *Q. ilex* extract are given in Table-1. At a concentration of 0.03 g/mL, the phenolic content was characterized by total phenols with 0.355 ± 0.004 mg gallic acid (GA)/g dry weight (DW), ortho-diphenols with 0.749 ± 0.014 mg GA/g DW, and flavonoids with 0.062 ± 0.006 mg CAT (catechin)/g DW. At a concentration of 0.06 g/mL, the phenolic composition exhibited the following measurements: total phenols amounted to 0.665 ± 0.013 mg GA/g DW; ortho-diphenols at 1.369 ± 0.038 mg GA/g DW; and flavonoids at 0.144 ± 0.005 mg CAT/g DW. At a concentration of 0.09 g/mL, the phenolic content was characterized by 0.929 ± 0.018 mg GA/g DW for total phenols, 1.877 ± 0.022 mg GA/g DW for ortho-diphenols, and 0.226 ± 0.006 mg CAT/g DW for flavonoids. There were statistically significant differences in the total phenolic content among the tested concentrations. Regarding antioxidant capacity, the *Q. ilex* extract prepared at 0.03 g/mL had a capacity of 3.340 ± 0.013 mmol Trolox/g DW, the 0.06 g/mL concentration had a capacity of 6.820 ± 0.118 mmol Trolox/g DW, and the *Q. ilex* extract prepared at 0.09 g/mL had a capacity of 9.205 ± 0.099 mmol Trolox/g DW. Significant differences were also found among all concentrations (p < 0.05) (Table-2). The *Q. ilex* extract was mainly composed of digalloyl-glucose, followed by GA (Table-2).

**Table-1:** Mean, standard deviation, and univariate effects of total phenolic (mg GA/g DW), ortho-diphenol (mg GA/g DW), and flavonoid (mg CAT/g DW) content and antioxidant capacity (mmol Trolox/g DW).

Concentration	Phenolic content			Antioxidant capacity
	
	Total phenolics	Ortho-diphenols	Flavonoids	FRAP
0.03 g/mL	0.355 ± 0.004	0.749 ± 0.014	0.062 ± 0.006	3.340 ± 0.013
0.06 g/mL	0.665 ± 0.013	1.369 ± 0.038	0.144 ± 0.005	6.820 ± 0.118
0.09 g/mL	0.929 ± 0.018	1.877 ± 0.022	0.226 ± 0.006	9.205 ± 0.099

GA=Gallic acid, DW=Dry weight, CAT=Catalase, FRAP=Ferric reducing antioxidant power

**Table-2:** Identification and quantification (mg/g extract) of phenolic compounds in the aqueous extract of *Quercus ilex*.

Peak	Rt (min)	λmax (nm)	[M-H] (m/z)	MS^2^	Tentative identification	Quantification (mg/g extract)
1	4.33	277	169	125 (100)	Gallic acid	3.23 ± 0.04
2	4.50	273	483	313 (43);313 (100);169 (23)	Digalloyl-glucose	9.10 ± 0.30
3	7.61	280	289	245 (100);203 (12)	(-) –Epicatequin	1.50 ± 0.07
4	8.27	276	387	207 (100);163 (53)	Medioresinol	0.62 ± 0.03
5	8.32	278	451	313 (100);169 (10)	Trigalloyl acid lactonized	0.58 ± 0.02
					Total phenolics compounds	15.10 ± 0.20

Rt=Retention time (min), λmax=Maximum absorption in the visible region (nm), [M-H]=Diprotonatedd ion (m/z), MS^2^=Mass fragmentation (m/z)

### General findings, food and drink intake, and organ weight

No behavioral changes, including environmental responses, such as reaction to stimuli (e.g., placing the palm of the hand above the cage), respiratory rate, social interaction with other animals in the same cage, dietary patterns, and exploratory behavior, were observed among the experimental groups. No animal scored four for the humane endpoints; therefore, the results suggest that *Q. ilex* extract did not interfere with animal welfare. Statistically significant differences were observed between G2 (HPV, water) and G6 (HPV, 0.09 g/mL), indicating that *Q. ilex* extract improved the animals’ well-being (Table-3). G1 (WT, water) (1.43%), G2 (HPV, water) (2.66%), G3 (WT, 0.09 g/mL) (3.50 %), and G5 (HPV, 0.06 g/mL) (0.99%) showed an increase in body weight over the experimental period. The ANOVA results showed statistically significant differences between G2 (HPV, water) and G4 (HPV, 0.06 g/mL) (p < 0.05) (Table-3). Table-3 presents the findings regarding food and beverage consumption, which indicated a trend toward increased consumption by transgenic mice (HPV). Relative organs’ weight is displayed in Table-4. Statistically significant differences were found in lung between G2 (HPV, water) and G4 (HPV, 0.03 g/mL), as well as between G2 (HPV, water) and G5 (HPV, 0.06 g/mL) (p < 0.05), indicating that the differences were significantly increased. In addition, in the liver, G6 (HPV, 0.09 g/mL) was significantly higher than G3 (WT, 0.09 g/mL) (p < 0.05). The groups that ingested *Q. ilex* extract also had a higher kidney weight. Statistically significant differences were found in the right kidneys between G2 (HPV, water) and G6 (HPV, 0.09 g/mL), as well as between G3 (WT, 0.09 g/mL) and G6 (HPV, 0.09 g/mL) (p < 0.05).

**Table-3:** Humane endpoints: body weight (g) at the start and end of the study, weight gain (%), average daily consumption of food (g), and drink (mL) during the experimental trial (mean ± standard deviation).

Group	Humane endpoints	Body weight		Weight gain (%)	Average daily consumption	
	
		Initial (g)	Final (g)		Food (g)	Drink (mL)
G1 WT water	0.73 ± 0.26	24.67 ± 2.87	25.01 ± 2.78	1.43 ± 2.36	3.46	4.07
G2 HPV water	1.02 ± 0.18^1^	22.78 ± 0.69	23.38 ± 0.66	2.66 ± 0.77^2^	4.19	6.21
G3 WT, 0.09 g/mL	0.78 ± 0.28	24.34 ± 3.85	25.13 ± 3.59	3.50 ± 3.03	3.44	4.75
G4 HPV 0.03 g/mL	0.87 ± 0.15	23.33 ± 1.61	23.54 ± 1.49	0.99 ± 3.95	4.08	6.20
G5 HPV 0.06 g/mL	0.85 ± 0.27	23.94 ± 1.82	23.19 ± 1.47	-3.00 ± 4.33	4.30	6.57
G6 HPV 0.09 g/mL	0.57 ± 0.16	24.78 ± 0.74	24.61 ± 0.67	-0.69 ± 0.94	4.29	6.96

1 Statistically different from G6 (p < 0.05).

2 Statistically different from G5 (p < 0.05). HPV=Human papillomaviruses, WT=Wildtype

**Table-4:** Relative organ weight (%) (mean ± standard deviation).

Organs	G1 WT water	G2 HPV water	G3 WT, 0.09 g/mL	G4 HPV 0.03 g/mL	G5 HPV 0.06 g/mL	G6 HPV 0.09 g/mL
Heart	0.40 ± 0.06	0.42 ± 0.09	0.44 ± 0.09	0.45 ± 0.06	0.50 ± 0.05	0.48 ± 0.03
Lung	0.58 ± 0.08	0.56 ± 0.06^1,2^	0.62 ± 0.06	0.70 ± 0.03	0.73 ± 0.05	0.66 ± 0.08
Spleen	0.34 ± 0.13	0.39 ± 0.08	0.36 ± 0.06	0.40 ± 0.05	0.43 ± 0.08	0.49 ± 0.13
Liver	3.92 ± 0.18	4.32 ± 0.27	3.74 ± 0.28^3^	4.45 ± 0.32	4.48 ± 0.23	4.60 ± 0.35
Right kidney	0.54 ± 0.06	0.53 ± 0.07^3^	0.53 ± 0.06^3^	0.57 ± 0.06	0.62 ± 0.05	0.64 ± 0.04
Left kidney	0.53 ± 0.06	0.60 ± 0.07	0.51 ± 0.09	0.57 ± 0.05	0.65 ± 0.06	0.61 ± 0.07

1 Significantly different from G4 (p < 0.05).

2 Significantly different from G5 (p < 0.05).

3 Significantly different from G6 (p < 0.05) HPV=Human papillomaviruses, WT=Wild-type

### Microhematocrit and serum biochemical parameters

Microhematocrit and serum biochemical parameters are displayed in Table-5. Statistically significant differences in total proteins were found between G4 (HPV, 0.03 g/mL) and G6 (HPV, 0.09 g/mL) (p < 0.05), as well as between G5 (HPV, 0.06 g/mL) and G6 (HPV, 0.09 g/mL) (p < 0.05). Urea values were higher in G5 (HPV, 0.06 g/mL), and statistically significant differences were found between G4 (HPV, 0.03 g/mL) and G5 (HPV, 0.06 g/mL) (p < 0.05).

**Table-5:** Microhematocrit, glucose, and serum biochemical parameters (mean ± standard deviation).

Parameters	G1 WT water	G2 HPV water	G3 WT 0.09 g/mL	G4 HPV 0.03 g/mL	G5 HPV 0.06 g/mL	G6 HPV 0.09 g/mL
Microhematocrit (%)	45.68 ± 0.99	46.05 ± 2.52	45.25 ± 3.81	44.95 ± 2.68	46.63 ± 2.31	44.63 ± 2.72
Glucose (mg/dL)	203.67 ± 20.76	170.83 ± 34.98	165.17 ± 20.98	174.33 ± 14.39	198.83 ± 32.71	170.67 ± 22.80
Albumin (g/L)	2.89 ± 0.41	2.69 ± 0.23	3.03 ± 0.42	2.86 ± 0.55	2.70 ± 0.20	3.04 ± 0.42
Alanine aminotransferase (U/L)	75.38 ± 17.84	61.03 ± 12.62	48.50 ± 17.81	122.73 ± 116.81	85.92 ± 23.94	90.78 ± 20.03
Total protein level (g/L)	45.12 ± 3.39	46.48 ± 5.59	47.18 ± 3.10	43.83 ± 2.831	43.34 ± 1.271	50.88 ± 1.95
Urea (mg/dL)	61.12 ± 8.95	62.77 ± 7.47	62.90 ± 11.70	71.83 ± 3.972	53.08 ± 6.55	67.50 ± 7.31

^1^Statistically different from G6 (p < 0.05). ^2^Statistically different from G5 (p < 0.05). HPV=Human papillomaviruses, WT=Wild-type

### Histology

#### HPV-induced lesions

**Figure-1:**
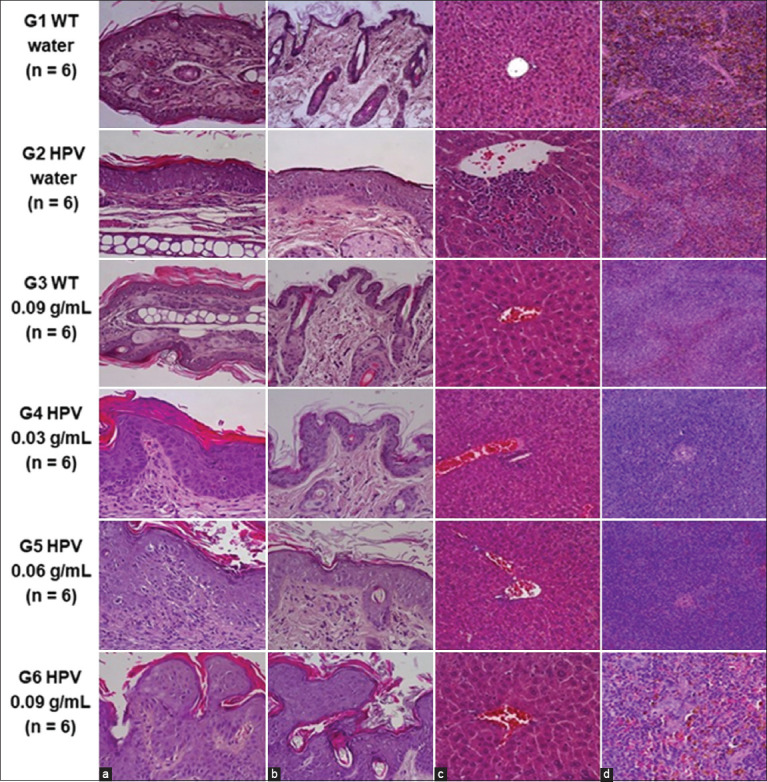
Histological analysis of (a) ear pavilion skin, (b) chest skin, (c) liver, and (d) spleen, in each experimental group. (a) WT groups showing normal ear pavilion skin (A-G1, A-G3), HPV groups showing papilloma (A-G6), carcinoma *in situ* (AG4), and hyperplasia (AG5) and small invasive carcinoma (A-G2); (b) WT groups showing normal skin (B-G1, B-G3), HPV groups showing small invasive carcinoma (B-G4), dysplasia (B-G5), papilloma (B-G6), and carcinoma *in situ* (B-G2). (c) WT groups showing normal liver (C-G1) with low-grade of congestion (C-G3), HPV groups showing inflammatory infiltrate (C-G2) and congestion (C-G2, C-G4, C-G5, and C-G6); (d) WT groups showing normal spleen (D-G1) with low grade of white pulp hyperplasia (D-G3), HPV groups showing normal spleen (G-G4, G-G6), and low grade of white pulp hyperplasia (G-G5) (40× H&E). HPV=Human papillomaviruses, WT=Wild-type.

Table-6 and Figures-1a and b summarize the results of histological analysis of the ear and chest skin. As expected, cutaneous lesions on the skin of the ear pavilion were observed only in the HPV groups, whereas all animals in the two WT groups exhibited normal auricular skin. Neoplastic lesions were recorded in all HPV-positive groups. Regarding the ear skin, there were statistically significant differences between G1 (WT, water) and G2 (HPV, water) in the parameters of dysplasia, inflammatory infiltrate, and hyperplasia (p < 0.05). In the skin samples of the chest, there were also statistically significant differences between G1 (WT, water) and G2 (HPV, water) (p < 0.05) in the parameter sebaceous hyperplasia. WT animals (G1 (WT, water) and G3 (WT, 0.09 g/mL)) exhibited normal chest skin (100%), thus confirming that the *Q. ilex* extract did not cause any histological skin lesions in WT animals. All control animals from G2 (HPV, water) exhibited histological lesions of sebaceous hyperplasia (100%), with statistically significant differences between G1 (WT, water) and G2 (HPV, water) (p < 0.05). Dysplasia was more frequent in G4 (HPV, 0.03 g/mL) (67%) and G6 (HPV, 0.09 g/mL) (50%), followed by G5 (HPV, 0.06 g/mL) (17%). Neoplastic lesions were found in all HPV groups. G4 (HPV, 0.03 g/mL) and G5 (HPV, 0.06 g/mL) had three recorded cases of carcinoma *in situ* (50%), whereas G2 (HPV, water) had four cases of carcinoma *in situ* (67%) and two cases of small invasive carcinoma (33%). In addition, a small invasive carcinoma was detected in G5 (HPV, 0.06 g/mL) (17%).

**Table-6:** Animals with histological cutaneous lesions in all experimental groups.

Histological cutaneous lesions	G1 WT water (%)	G2 HPV water (%)	G3 WT 0.09 g/mL (%)	G4 HPV 0.03 g/mL (%)	G5 HPV 0.06 g/mL (%)	G6 HPV 0.09 g/mL (%)
**Ear pavilion lesions**						
Dysplasia	0 (0)^1^	6 (100)	0 (0)	4 (67)	4 (67)	5 (83)
Papilloma	0 (0)	1 (17)	0 (0)	1 (17)	1 (17)	3 (50)
Sebaceous hyperplasia	0 (0)	4 (67)	0 (0)	6 (100)	2 (33)	5 (83)
Inflammatory infiltrate	0 (0)^1^	6 (100)	0 (0)	6 (100)	4 (67)	6 (100)
Hyperplasia						
Simple						
Focal	0 (0)	0 (0)	0 (0)	0 (0)	0 (0)	0 (0)
Diffuse	0 (0)^1^	6 (100)	0 (0)	5 (83)	2 (33)	5 (83)
Papillary						
Focal	0 (0)	2 (33)	0 (0)	2 (33)	2 (33)	4 (67)
Diffuse	0 (0)	0 (0)	0 (0)	0 (0)	1 (17)	1 (17)
Papillomatosis						
Focal	0 (0)	4 (67)	0 (0)	3 (50)	1 (17)	4 (67)
Diffuse	0 (0)	1 (17)	0 (0)	1 (17)	2 (33)	1 (17)
Carcinoma						
Carcinoma *in situ*	0 (0)	6 (100)	0 (0)	5 (83)	3 (50)	5 (83)
Small-invasive carcinoma	0 (0)	4 (67)	0 (0)	3 (50)	3 (50)	0 (0)
Invasive carcinoma	0 (0)	1 (17)	0 (0)	1 (17)	0 (0)	0 (0)
**Chest skin lesions**						
Dysplasia	0 (0)	3 (50)	0 (0)	4 (67)	1 (17)	3 (50)
Sebaceous hyperplasia	0 (0)^1^	6 (100)	0 (0)	5 (83)	4 (67)	5 (83)
Inflammatory infiltrate	0 (0)	2 (33)	0 (0)	1 (17)	2 (33)	3 (50)
Hyperplasia						
Simple						
Focal	0 (0)	0 (0)	0 (0)	1 (17)	0 (0)	0 (0)
Diffuse	0 (0)	3 (50)	0 (0)	4 (67)	0 (0)	1 (17)
Papillary						
Diffuse	0 (0)	3 (50)	0 (0)	1 (17)	3 (50)	4 (67)
Focal	0 (0)	2 (33)	0 (0)	1 (17)	1 (17)	1 (17)
Papillomatosis						
Diffuse	0 (0)	0 (0)	0 (0)	0 (0)	1 (17)	0 (0)
Papilloma						
Inverted papilloma	0 (0)	0 (0)	0 (0)	2 (33)	2 (33)	1 (17)
Exophytic papilloma	0 (0)	0 (0)	0 (0)	1 (17)	3 (50)	3 (50)
Carcinoma						
Carcinoma *in situ*	0 (0)	4 (67)	0 (0)	3 (50)	3 (50)	5 (83)
Small-invasive carcinoma	0 (0)	2 (33)	0 (0)	0 (0)	1 (17)	0 (0)

1 Significantly different from the G2 (p < 0.05). HPV=Human papillomaviruses, WT=Wildtype

### Histological analysis of internal organs

Table-7 and in Figures-1c and d summarizes the histological results of the lungs, liver, spleen, and kidneys. In the liver, there were statistically significant differences between G1 (WT, water) and G2 (HPV, water) (p < 0.05) in parameter congestion. Statistically significant differences were observed in the parameter diffuse white pulp hyperplasia between the G1 (WT, water) and G2 (HPV, water) (p < 0.05) in the spleen. Hyperplasia was observed in all mice from G2 (HPV, water) (100%), in four mice from G3 (WT, 0.09 g/mL) (67%), in two mice from G4 (HPV, 0.03 g/mL) (33%), and in one mouse from G5 (HPV, 0.06 g/mL) (17%) and G6 (HPV, 0.09 g/mL) (17%).

**Table-7:** Number of animals (%) with histological lesions in the lungs, liver, spleen, and kidneys in all experimental groups.

Histological lesions	G1 WT water (%)	G2 HPV water (%)	G3 WT, 0.09 g/mL (%)	G4 HPV 0.03 g/mL (%)	G5 HPV 0.06 g/mL (%)	G6 HPV 0.09 g/mL (%)
**Lungs**						
Mononucleated inflammation intensity	0 (0)	2 (33)	1 (17)	0 (0)	0 (0)	0 (0)
Atelectasis	0 (0)	1 (17)	0 (0)	0 (0)	2 (33)	0 (0)
Emphysema	0 (0)	3 (50)	3 (50)	0 (0)	1 (17)	0 (0)
Bleeding	0 (0)	2 (33)	2 (33)	0 (0)	0 (0)	0 (0)
Airways desquamation	0 (0)	1 (17)	0 (0)	0 (0)	0 (0)	0 (0)
Hyalinization arteriolar	0 (0)	1 (17)	0 (0)	0 (0)	0 (0)	0 (0)
Hyperemia	0 (0)	4 (67)	4 (67)	2 (33)	1 (17)	2 (33)
Hemorrhage	0 (0)	2 (33)	1 (17)	0 (0)	0 (0)	0 (0)
**Liver**						
Vacuolar	1 (17)	0 (0)	0 (0)	0 (0)	0 (0)	0 (0)
Inflammatory infiltrate						
Periportal	0 (0)	1 (17)	0 (0)	1 (17)	0 (0)	0 (0)
Centrilobular	0 (0)	3 (50)	0 (0)	0 (0)	0 (0)	0 (0)
Midzonal	1 (17)	1 (17)	1 (17)	3 (50)	1 (17)	0 (0)
**Spleen**						
Diffuse white pulp: hyperplasia	0 (0)^1^	6 (100)	4 (67)	2 (33)	1 (17)	1 (17)
Expansion of splenic red pulp: congestion	0 (0)	0 (0)	1 (17)	0 (0)	0 (0)	0 (0)
**Kidney**						
Inflammatory infiltrate	1 (17)	6 (100)	2 (33)	4 (67)	2 (33)	6 (100)
Hydronephrosis	0 (0)	3 (50)	0 (0)	1 (17)	1 (17)	1 (17)
Hyperemia	0 (0)	1 (17)	0 (0)	0 (0)	0 (0)	1 (17)
Vascular ectasia	0 (0)	3 (50)	3 (50)	0 (0)	0 (0)	0 (0)

1 Significantly different from that of G2 (p < 0.05). HPV=Human papillomaviruses, WT=Wild-type

### Liver and kidney oxidative stress

**Figure-2:**
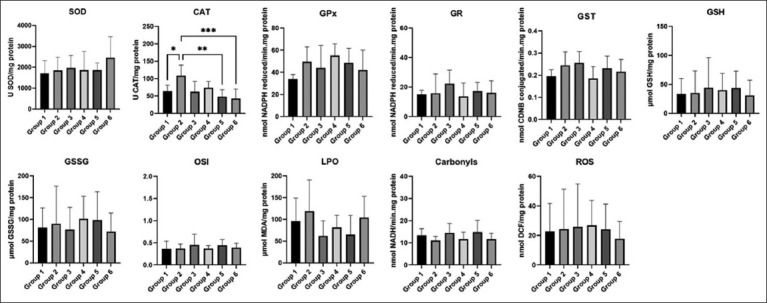
Liver oxidative stress. Statistically significant differences in the CAT marker between G1 (WT, water) and G2 (HPV, water), G2 (HPV, water) and G5 (HPV, 0.06 g/mL) and between G2 (HPV, water) and G6 (HPV, 0.09 g/mL) (*p* < 0.05). Cu/Zn-SOD (U SOD/mg protein); CAT (U CAT/mg protein); GPx (nmol NADPH reduced/min.mg protein); GR (nmol NADPH reduced/min.mg protein); GST (nmol CDNB conjugated/min.mg protein); GSH (μmol GSH/mg protein); GSSG (μmol GSSG/mg protein); OSI (GSH/GSSG); LPO (μmol MDA/mg protein); Carbonyls (CO) (nmol nicotinamide adenine dinucleotide (NADH)/min.mg protein) and ROS (nmol fluorescent dichlorofluorescein (DCF)/mg protein). HPV=Human papillomaviruses, WT=Wildtype, CAT=Catalase, SOD=Superoxide dismutase, GPx=Glutathione peroxidase, GR=Glutathione reductase, GST=Glutathione S-transferase, GSH=Glutathione, OSI=Oxidative stress index, MDA=Malondialdehyde, LPO=Lipid peroxidation, ROS=Reactive oxygen species, CDNB=1-chloro-2,4-dinitrobenzene.

**Figure-3:**
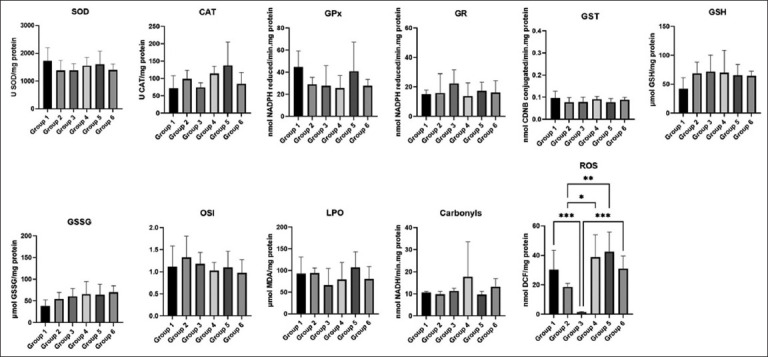
Kidney oxidative stress. Statistically significant differences in the ROS marker between G1 (WT, water) and G3 (WT, 0.09 g/mL); G2 (HPV, water) and G4 (HPV, 0.03 g/mL); G2 (HPV, water) and G5 (HPV, 0.06 g/mL); G3 (WT, 0.09 g/mL); and G6 (HPV, 0.09 g/mL) (*p* < 0.05). Cu/Zn-SOD (U SOD/mg protein); CAT (U CAT/mg protein); GPx (nmol NADPH reduced/min.mg protein); GR (nmol NADPH reduced/min.mg protein); GST (nmol CDNB conjugated/min.mg protein); GSH (μmol GSH/mg protein); GSSG (μmol GSSG/mg protein); OSI (GSH/GSSG); LPO (μmol MDA/mg protein), CO (nmol NADH/min.mg protein); ROS (nmol DCF/mg protein). HPV=Human papillomaviruses, WT=Wild-type, CAT=Catalase, SOD=Superoxide dismutase, GPx=Glutathione peroxidase, GR=Glutathione reductase, GST=Glutathione S-transferase, GSH=Glutathione, OSI=Oxidative stress index, MDA=Malondialdehyde, LPO=Lipid peroxidation, ROS=Reactive oxygen species, CDNB=1-chloro-2,4-dinitrobenzene.

Regarding the analysis of liver oxidative stress, statistically significant differences in the CAT marker were found between G1 (WT, water) and G2 (HPV, water), G2 (HPV, water) and G5 (HPV, 0.06g/mL), and between G2 (HPV, water) and G6 (HPV, 0.09 g/mL) (Figure-2) (p < 0.05). In the analysis of renal oxidative stress, there appears to be a tendency for higher levels of reactive oxygen species (ROS) markers to be found between G1 (WT, water) and G3 (WT, 0.09 g/mL), G2 (HPV, water) and G4 (HPV, 0.03 g/mL), G2 (HPV, water) and G5 (HPV, 0.06 g/mL), and between G3 (WT, 0.09 g/mL) and G6 (HPV, 0.09 g/mL) (Figure-3) (p < 0.05).

## Discussion

PVs induce warts and condylomas in animals and humans, showing species specificity across mammals, birds, fish, and reptiles [1, 2]. PV infections are widespread among various animal species globally, significantly impacting animal and livestock production and indirectly affecting human health [18]. Hence, exploring sustainable preventive measures using the One Health approach is crucial [19]. Researchers have long studied natural compounds due to their remarkable chemical diversity, and they are considered valuable sources of bioactive compounds with potential preventive and therapeutic applications in several diseases, including cancer [19, 20].

Acorns contain flavonoids, phenolic acids, and tannins that are vital for sustaining beneficial antioxidant levels in the body, thereby preventing diseases such as cancer, diabetes, and cardiovascular diseases [58]. They have several properties, including antioxidants [59], antimicrobials [60], anti-inflammatory [61], antidiabetics [62], hepatoprotective [63], antiobesity [64], anticancer [65], and anti-neurodegenerative [66]. Therefore, our objective was to evaluate the *in vivo* chemopreventive activity of an infusion extract of *Q. ilex* in a transgenic mouse model of HPV16. Chemical analysis of our *Q. ilex* infusion extract demonstrated the presence of GA, digalloyl-glucose, epicatequin, medioresinol, and trigalloyl acid lactonized. GA has been identified as an inhibitor of carcinogenesis in animal models and *in vitro* cancer cell lines, such as KATO III, COLO 205, 3T3L1, DUL145, 22Rv1, Calu-6, A459, HeLa, U87, U251n, HL-60, and HT-29 [67]. Regarding the phenolic profile, five compounds were identified and quantified, most of which were previously identified in acorn samples in the literature [68, 69]. Compound 5 (trigalloyl acid lactonized) has also been identified in other fruits [70].

In this research, the human endpoints were established with the aim of minimizing animal pain during experimental tests, as described by Nemzek *et al*. [71]. Our data, which were recorded weekly regarding the evaluation of humane endpoints, demonstrated no behavioral or morphological changes in the mice. Furthermore, the human endpoint scores were lower in G6 mice (HPV, 0.09 g/mL) compared with G2 (HPV, water). However, concerning the global mean of human endpoint parameters, a statistically significant decrease was observed between G2 (HPV, water) and G6 (HPV, 0.09 g/mL). This indicates that at the maximum concentration, keratosis in the ears and muzzle is reduced, as well as an improved appearance of the protective region around the eyes. No deaths, behavioral changes, or other clinical signs of discomfort were observed.

Furthermore, the mean body weight of the HPV-transgenic animals was significantly lower than that of the WT animals. This result has been previously documented [72] and is consistent with the cachexia observed in this animal model [72]. All animals showed an increase in weight gain, except for those in groups G4 (HPV, 0.03 g/mL) and G6 (HPV, 0.09 g/mL). Specifically, weight gain in G4 (HPV, 0.03 g/mL) was statistically lower than that in G2 (HPV, water). However, it is within the normal range for body weight [73]. Another hypothesis suggests that body weight is related to increased hydration, potentially explaining the lower body weight observed in transgenic animals [74]. Transgenic animals exhibit higher food and water intake compared to WT mice. The prevalent cachexia observed, which is characterized by weight loss and accelerated metabolism, may prompt quicker dehydration and, thus, increased water intake to compensate for fluid loss [72, 75].

Furthermore, the biological alteration of the skin barrier in these animals, resulting from subsequent transgenesis processes, contributes to a thinner skin barrier, potentially influencing their hydration needs [76]. The increased food consumption observed in transgenic mice could be attributed to a form of energy compensation; in other words, cachexia in transgenic animals elevates basal metabolism, consequently heightening their energy requirements [77]. Consequently, these animals may exhibit increased food consumption as a compensatory mechanism to meet the escalated energy expenditure. Conversely, cachexia is linked to chronic inflammatory processes that can upregulate the production of proinflammatory cytokines [78]. These cytokines, in turn, may elicit responses in the central nervous system, increasing appetite and food intake [79]. Inflammation may also contribute to immune suppression and tumor persistence, and anti-inflammatory therapy was previously shown to enhance the activity of cytotoxic T lymphocytes, leading to decreased tumor progression in this animal model [80].

In a general way, the rise in all relative organs, such as the heart, lung, spleen, liver, and kidneys, among the HPV groups administered with *Q. ilex* extract may suggest an enhanced metabolic response, potentially linked to various processes, such as heightened energy metabolism or increased cellular activity [81]. Elevated metabolic rates can lead to increased production of free radicals and oxidative stress, thereby prompting increased activity of antioxidant systems [82]. Alternatively, the increase in the weight of all organs could represent an adaptive or compensatory reaction of the body to *Q. ilex* extract exposure, as the body seeks to manage the extract components or their effects [83]. Indeed, while the escalation in the weight of all organs may suggest a physiological response, it is crucial to consider the possibility that this increase could signify stress or toxicity within the organs in reaction to *Q. ilex* extract. More specifically, the relative weight of the liver and spleen was greater in transgenic mice, which was anticipated due to the inflammatory processes documented in the literature associated with transgenesis [33]. When genetic alterations occur in an organism, such as the insertion of genetic material, they can trigger an immunological response, resulting in inflammation in various tissues, including the liver and spleen, which are vital to the immune system. Hence, the introduction of new genetic material into cells or tissues can induce an inflammatory response, which is an integral component of the body’s immune reaction [84]. However, administration of *Q. ilex* infusion seemed to corroborate the increase in liver and spleen weight. Overall, the observed increase in organ weight across all three concentrations of the extract in HPV mice suggests a systemic effect on the mice’s bodies, influencing all of the collected and analyzed organs. This phenomenon can be attributed to the biological impact of the extract’s bioactive components at the studied concentrations.

There were no statistically significant differences in the microhematocrit levels between the groups, suggesting that the infusion extract of *Q. ilex* does not induce anemia. The determination of microhematocrit is a precise analysis for the general study of anemia [85]. Glucose levels decreased in WT and HPV mice treated with *Q. ilex* extract (0.09 g/mL) but increased in HPV animals treated with 0.03 and 0.06 g/mL. This result may indicate the effect of *Q. ilex* extract at a concentration of 0.09 g/mL on blood glucose regulation, potentially increasing glucose levels. It may also suggest a differential response to treatment depending on the health status of the mice. In WT and HPV mice, a concentration of 0.09 g/mL had a hypoglycemic effect. This suggests that compounds in *Q. ilex* extract affect glucose metabolism by either increasing glucose uptake by cells or improving insulin sensitivity [86]. In HPV mice, the other concentrations have a hyperglycemic effect, suggesting that glucose regulation in these animals is altered or counterbalanced by the presence of the disease, leading to increased glucose levels. It is known that viral infection of cells requires increased glucose uptake for viral replication and production [87]. Therefore, in the context of HPV infection, it is reasonable to assume that infected cells are increasing their glucose demand, which could result in hyperglycemia in HPV mice. Therefore, this result can be attributed to the altered metabolic response of these animals to viral infection. Viral infection may increase the demand for glucose in infected cells, and *Q. ilex* extract may exacerbate this effect, resulting in higher blood glucose levels. Xu *et al*. [62] reported that ethanolic acorn extract protected diabetic rats by reducing blood glucose, increasing insulin secretion, and alleviating weight loss. Albumin is a blood plasma protein. Low levels can result from kidney and liver disease, inflammation, or infections, whereas high levels are typically associated with dehydration or severe diarrhea [88, 89]. Albumin levels appear to have increased in the *Q. ilex* extract groups compared with the control groups, hinting at possible dehydration in mice. ALT is a marker of liver function [89]. Elevated ALT levels were noted in certain groups treated with *Q. ilex* extract, suggesting potential liver damage or dysfunction associated with treatment. The groups treated with *Q. ilex* extract at 0.09 g/mL exhibited higher levels of total proteins than the control group. Elevated blood total protein levels are often associated with dehydration or inflammation, consistent with the results for albumin [90]. Elevated urea levels were observed in the *Q. ilex* extract-treated groups, indicating a potential impact of *Q. ilex* extract on kidney function. High urea levels could indicate kidney injury or disease [91]. Based on the histological analysis of the ears and chest skin, we anticipated that WT mice would not exhibit lesions, as they are considered healthy animals. All HPV-exposed mice exhibited notorious epidermal hyperplasia in the ear pavilion skin, some with papillomatosis, and sebaceous hyperplasia. Thus, more severe lesions were registered in G2 (HPV, water), two benign (papillomas) and one invasive carcinoma. Considering the severity of cutaneous lesions in chest skin, specifically neoplastic development, the worst group was G2 (HPV, water) with two carcinomas, followed by G5 (HPV, 0.06 g/mL) with one carcinoma. Globally, regarding all cutaneous lesions from the two regions contemplated in the study, G2 (HPV, water), G4 (HPV, 0.03 g/mL), and G6 (HPV, 0.09 g/mL) showed more aggressive lesions, indicating that *Q. ilex* extract taken at one concentration has a preventive effect against chest skin lesions, namely, in neoplastic progression. In 2019, Lee *et al*. [92] demonstrated that acorn husk extract improved the progression of atopic dermatitis-like lesions, including swelling, induced by oxazolone in Balb/c mouse ears. Among transgenic mice, *Q. ilex* extract did not significantly alter the percentage of histological lesions in internal organs, including the inflammatory hepatic and splenic lesions typically described in this mouse strain. In terms of hepatic lesions, G2 (HPV, water) exhibited a greater number of lesions as anticipated, affecting a larger proportion of mice within the group. Following this, G5 (HPV, 0.03 g/mL) exhibited a similar pattern. Conversely, G6 (HPV, 0.09 g/mL) had the most favorable outcomes, which can be attributed to the therapeutic effect and absence of toxicological effects of *Q. ilex* extract and its concentration. Regarding the kidneys, G2 (HPV, water) was the most adversely affected group. All animals in group 2 (HPV, water) exhibited infiltrates as well as hydronephrosis, however, only one case presented with hyaline cylinders Subsequently, G6 (HPV, 0.09 g/mL) and G3 (WT, 0.09 g/mL) exhibited similar renal pathological patterns. The 0.06 g/mL concentration decreased the perception of histological lesions, such as hyperplasia, dysplasia, and carcinoma. Once more, from a histopathological perspective, a single concentration exhibited beneficial effects. Regarding oxidative stress in the liver, *Q*. *ilex* extract appears to exhibit antioxidant activity in the liver, as evidenced by the increased activity of antioxidant enzymes such as SOD, GPx, GR, and GST in certain groups (e.g., G3 [WT, 0.09 g/m] and G4 [HPV, 0.03 g/mL]). This suggests that *Q. ilex* extract protects the liver against oxidative stress by enhancing its antioxidant capacity. However, the effects of *Q. ilex* extract on the liver appear to vary depending on the concentration of the extract administered. For instance, different groups displayed disparate antioxidant responses, with certain concentrations, such as 0.09 g/mL, showing an increase in antioxidant activity and 0.03 g/mL showing a decrease. In addition, the presence of HPV in the experimental groups seems to influence the results. For example, G2 (HPV, water) exhibited enhanced antioxidant activity against CAT and LPO, indicating a potential virus-specific response. Moreover, some groups demonstrated increased oxidative damage, as evidenced by elevated carbonyl and ROS levels in G4 (HPV, 0.03 g/mL) and G5 (HPV, 0.06 g/mL). This suggests that *Q. ilex* extract may not effectively prevent oxidative damage at all concentrations or experimental conditions. Regarding oxidative stress in the kidneys, there appears to be variation in antioxidant activity among the different groups. The concentration of 0.06 g/mL showed higher activity in antioxidant enzymes such as CAT, GPx, and GST, whereas the other concentrations exhibited lower activity in several of these enzymes. Once again, the presence of HPV seems to influence the results, as observed in G5 (HPV, 0.06 g/mL), which displayed increased antioxidant activity in some enzymes (SOD, CAT) and greater oxidative damage (ROS). The different groups also demonstrated variation in the levels of oxidative damage, with G4 (HPV, 0.03 g/mL) exhibiting greater carbonyl formation and G5 (HPV, 0.06 g/mL) exhibiting higher ROS formation. This suggests that different concentrations of *Q. ilex* extract exert different effects on oxidative damage to the kidneys. The findings regarding oxidative stress in both the liver and kidneys indicate that *Q. ilex* extract may have diverse effects, encompassing antioxidant activity, oxidative damage, and potential interactions with HPV.

## Conclusion

Administration of *Q. ilex* extract resulted in a noteworthy increase in relative organ weight among HPV transgenic animals, indicating a biological response to the tested concentrations. Moreover, skin lesions were reduced in animals treated with the 0.06 and 0.09 g/mL extract. These findings suggest the potential chemopreventive effects of *Q. ilex* extract, particularly against the progression of neoplastic skin lesions. The presence of bioactive compounds identified in the extract, such as GA and epicatequin, known for their antioxidant activity and chemopreventive potential in the previous studies, provides a plausible biological basis for these observed effects. Hence, we conclude that under these conditions and concentrations, Q. *ilex* extract is safe and does not compromise animal welfare. Future investigations can be conducted using the isolates from the extract to determine which are responsible for the identified effects.

## Authors’ Contributions

BMF and PAO: Conceptualization. BMF, AIFR, MJP, MJN, HV, CVN, IG, ANB, MD, LB, LG, MMSMB, LF, CV, RM, RMGDC, and PAO: Investigation. BMF: Writing – original draft. AIFR, MJP, MJN, HV, CVN, IG, ANB, MD, LB, LG, MMSMB, LF, CV, RM, RMGDC, and PAO: Writing – review and editing. RM, RMGDC, and PO: Supervision. All authors have read and approved the final manuscript.
